# A cellular star atlas: using astrocytes from human pluripotent stem cells for disease studies

**DOI:** 10.3389/fncel.2013.00025

**Published:** 2013-03-14

**Authors:** Robert Krencik, Erik M. Ullian

**Affiliations:** Departments of Ophthalmology and Physiology, Neuroscience Program, University of CaliforniaSan Francisco, CA, USA

**Keywords:** human stem cells, astrocytes, RASopathies, disease models, synaptogenic proteins, neurological disorders, regenerative medicine, developmental disorders of the brain

## Abstract

What roles do astrocytes play in human disease?This question remains unanswered for nearly every human neurological disorder. Yet, because of their abundance and complexity astrocytes can impact neurological function in many ways. The differentiation of human pluripotent stem cells (hPSCs) into neuronal and glial subtypes, including astrocytes, is becoming routine, thus their use as tools for modeling neurodevelopment and disease will provide one important approach to answer this question. When designing experiments, careful consideration must be given to choosing paradigms for differentiation, maturation, and functional analysis of these temporally asynchronous cellular populations in culture. In the case of astrocytes, they display heterogeneous characteristics depending upon species of origin, brain region, developmental stage, environmental factors, and disease states, all of which may render experimental results highly variable. In this review, challenges and future directions are discussed for using hPSC-derived astroglial progenitors and mature astrocytes for neurodevelopmental studies with a focus on exploring human astrocyte effects upon neuronal function. As new technologies emerge to measure the functions of astrocytes *in vitro* and *in vivo*, there is also a need for a standardized source of human astrocytes that are most relevant to the diseases of interest.

## Introduction

With the recent technological advances for astrocyte manipulation, generation, purification, and functional analyses in normal and diseased states, there is a need for standardization and optimization of the experimental system. In regards to this review, the system of interest is the differentiation of astrocytes from human pluripotent stem cells (hPSCs), either embryonic stem cells (ESCs) or induced pluripotent stem cells (iPSCs). During neurodevelopmental studies, it would be ideal to compare cell differentiation of control and experimental groups beginning with limited differences except for the variable of interest. Inevitably, epigenetic variations will exist within the hPSC lines prepared from different cellular origins (Kim et al., 2010) and those cultured with different methods (Nazor et al., 2012; Tomoda et al., 2012), all of which may lead to altered differentiation potentials. Also, genetic variability will always occur among human cellular sources, which has led to the design of methods for generating isogenic control hPSC lines through genetic correction technology (Zwaka and Thomson, 2003; Hockemeyer et al., 2009, 2011). These technologies (e.g., homologous recombination, zinc finger nucleases, and TALENS) are designed to target and replace a mutation of interest with wild-type sequence while keeping the rest of the genome unmodified. In the subsequent stage of directed neural induction and differentiation of hPSCs into neuroepithelia/neural stem cells (NSCs), multiple protocols have been designed with varying environmental factors and techniques which can alter the developmental timing and identity of the final cell type of interest [for example, the generation of CNS neural cells (Zhang et al., 2001) vs. PNS (Lee et al., 2007)]. Although variability has the advantage of masking non-specific phenotypes in disease models, and is also a better representation of variation in nature, it may lead to false positive experimental results when using a low number of replicates. While keeping in mind this heterogenous source material, we will focus this review on examining the variability and heterogeneity that occurs between the stages from hPSC-derived NSCs to astrocytes, and discuss the advantages and disadvantages of using an *in vitro* system to enumerate major experimental variables that should be taken into account when designing disease-related studies.

For which neurological diseases is an examination of astrocyte function relevant? There are numerous methods to mimic disease states in cultured astrocytes including scratch assays (Yang et al., 2012), mechanical stretch (Wanner et al., 2008), and treatments with inflammatory factors (Falsig et al., 2004), but the main benefit of using patient-specific iPSCs is to study specific disease-causing genetic mutations. The most obvious disorder to target is Alexander Disease, which is referred to as a “primary astrocyte disease” because it is caused by mutations of the semi-specific astrocyte protein GFAP, but also eventually leads to damage in oligodendrocytes and neurons through yet unknown mechanisms (Messing et al., 2012). On the other hand, it is becoming clear that many, if not all, neurodevelopmental and neurodegenerative diseases may be directly or indirectly affected by glial function (Molofsky et al., 2012; Verkhratsky et al., 2012). Whether the observed astrocytic phenotypes are disease-specific or generic consequences of a stressed “reactive” astrocyte (referred to here as astrogliosis) that contributes downstream to neighboring cells is a major question that should be examined in each case. For example, it has been observed in some amyotrophic lateral sclerosis models that astrocytes either secrete toxic factors [i.e., lipocalin 2 (Bi et al., 2013)] or have a deficiency in providing support to motoneurons, leading to neuronal degeneration. Whether these factors are the main cause of motoneuron loss and how they specifically affect these neurons is still not clear (Sica, 2012; Phatnani et al., 2013). In the case of neurodevelopmental disorders, an altered timing of astrocyte differentiation likely leads to changes in the number of adult astrocytes and/or in their impact upon neurons, as described in more detail below. For the purposes of this review, we will provide examples for experimentation using one of the most common classes of neurodevelopmental disorders that are likely affected by both developmental and functional changes in neural cells. These syndromes are commonly referred to as “RASopathies” because they all involve alterations in the Ras/MAPK signaling pathway and lead to mental impairments among other phenotypes (Tidyman and Rauen, 2009). Mouse models have shown that astrocyte progenitors have an accelerated development and/or proliferation in a number of these syndromes including Noonan syndrome (Gauthier et al., 2007), Neurofibromatosis-1 (Hegedus et al., 2007), Costello syndrome (Paquin et al., 2009), and cardiofaciocutaneous syndrome (Li et al., 2012; Tien et al., 2012), though the astrocyte-specific functional consequence on neurons in these contexts, especially in a human cellular system, is unknown.

What phenotypes should be examined? There are at least three major levels of cellular examination that can be addressed when comparing diseased and control astrocytes; (1) intrinsic changes within an individual cell such as gene expression and cell signaling, (2) population networks that include heterogeneous cell types and long range coupling, and (3) extrinsic factors released from astrocytes that affect other cell types including neurons, oligodendrocytes, microglia, or those that make up the vasculature. These levels are also temporally dynamic during differentiation and the functional consequences may depend on development, brain region, and environmental conditions (Zhang and Barres, 2010; Oberheim et al., 2012; Theis and Giaume, 2012). In some cases the appropriate choice of analysis is obvious when the specific cause of disease is known, i.e., astrocytes from an ALS model (mutant TDP-43) iPSC lines have an increased expression and mislocalization of TDP-43 protein (Serio et al., 2013), and astrocytes from Alzheimer's disease models (sporadic cases and mutant APP) have Aβ oligomer accumulation (Kondo et al., 2013). Though for the majority of other cases, finding a disease-related phenotype may take various large scale profiling methods. Below, using examples with RASopathy-specific cells, we will discuss the advantages and shortcomings of utilizing hPSC-derived astrocytes to study specific functional aspects both intrinsically and upon other cell types. Ultimately, the phenotypes should be confirmed in an *in vivo* environment with methods such as transplantation of the human astrocytes into rodent or primate brain.

## Consequences of fitting a stellar universe in a dish

**Figure 1:**
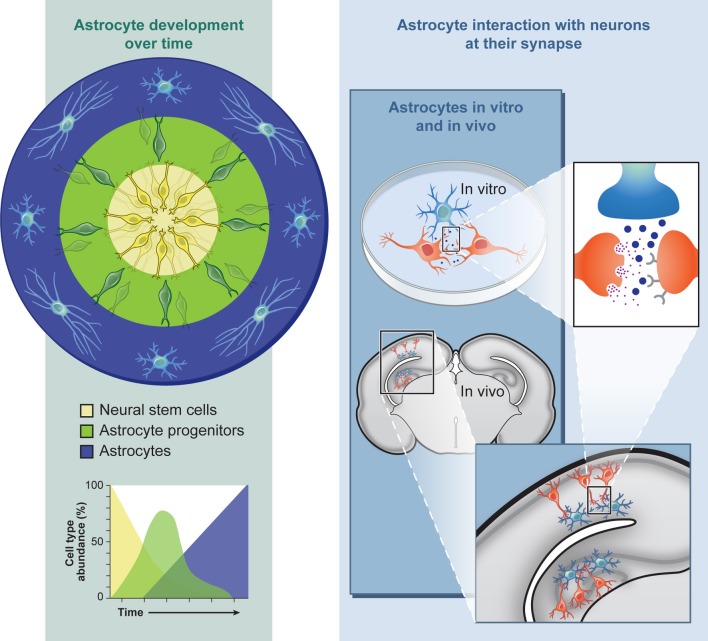
**Astroglial differentiation from human pluripotent stem cells allows for the investigation into neurodevelopmental and functional aspects of neurological and neurocognitive disorders in culture or after engraftment into intact nervous tissue**.

One major advantage of studying *in vitro* astrocyte progenitor differentiation from NSCs in a culture system is that the intrinsic developmental order (neurons, then glia) and timing (several months for human) correlates with *in vivo* development, due to a combination of transcriptional and epigenetic regulations (Sauvageot and Stiles, 2002; Okano and Temple, 2009). For developmental studies, this extended temporal program allows for human cellular profiling during differentiation at the gene expression (qPCR, microarray, RNA-seq), protein (immunocytochemistry, Western, proteomics), and epigenetic (methylation, histone modifications) levels at discrete stages (Krencik and Zhang, 2006). During RASopathy studies, this profiling could be used to determine whether abnormal Ras/MAPK signaling cause temporal shifts in astrogliosis, as observed in mouse models, and these data sets can provide baselines for comparisons after genetic or drug screening with the aim of modifying the abnormal developmental timing back to control levels. For example, cells may be acutely or chronically treated with compounds safe for clinical trials including inhibitors of farnesyl transferase, MEK, and ERK (Rauen et al., 2011), or expression levels may be hampered with siRNA technologies. One should bear in mind that one major drawback is that the population of neural progenitors derived from hPSCs are non-synchronous, e.g., there will always be a mixture of cells at slightly different stages of development (Figure 1). On the other hand, this phenomenon may have some silver linings. For example, three-dimensional differentiation of neuronal cultures in a cluster generates cells intrinsically organized in a spatial and temporal polarization that mimics radial glial and neuronal layering in the cortex (Eiraku et al., 2008), potentially producing a more accurate model of *in vivo* development. This structural recapitulation may also occur during astroglial differentiation although it has not been extensively investigated. With an *in vitro* system it is possible to recapitulate normal development by examining the temporal expression pattern of progenitor (NFIA, S100B) and more mature (GFAP) markers over time (Krencik et al., 2011). The scarcity of astrocyte markers does not yet allow for more precise examination of subtypes that occur throughout the CNS (one likely exemption are regionally-specific developmental transcription factors), but it may be possible to recapitulate the distinct morphological characteristics displayed by human astrocytes *in vivo*, which depends on their cortical location (Oberheim et al., 2009).

Perhaps the most important pressing issues in this field are how to properly identify a mature astrocyte and how to standardize this definition between research laboratories. GFAP has long been the gold standard as an astrocyte marker (Eng et al., 2000), even though its levels change during development, aging, and stress. At what point should a glial progenitor cell be termed an astrocyte using this marker? For immunocytochemical analysis, the appearance of GFAP protein is commonly used since there are many commercially available antibodies that consistently work well and the cellular protein content is highly abundant. Unfortunately, during early stages of the hPSC differentiation process, GFAP can be observed diffusely throughout the cells with high antibody concentrations and high exposure times, then gradually localizes in a filamentous pattern while becoming more intense (Krencik, pers. observation), thus the identification of an astrocyte using GFAP as a marker is not absolute. Other proteins used as markers also display shifting localization as the cells mature, for example CD44 localizes in a punctuate/ruffled manner after the receptor inserts into the cell surface membrane. Thus, standards for identifying onset of these markers may be unknowingly disparate among research groups that have thus far generated hPSC-derived astroglial progenitors for studies (Krencik et al., 2011; Gupta et al., 2012b; Juopperi et al., 2012; Serio et al., 2013; Shaltouki et al., 2013; Wang et al., 2013), leading to examination of cells at different developmental stages, levels of astrogliosis, or may even falsely identifying non-neural contaminating cells which can appear in this culture system (Krencik and Zhang, 2011). One suggestion for standardizing identification is to first measure primary astrocyte cultures that usually contain GFAP+ and GFAP- cells to determine maximum and minimum cutoffs during imaging, and then assemble information on the relative intensity of filamentous GFAP for each individual cell in an imaging field. Unfortunately, other astrocyte-specific markers such as Glt-1 and Aldh1L1 are more difficult to use for immunochemistry (Krencik, pers. observation) and less is known about their expression in human cells, yet tools based on these markers are effective reporters in mouse transgenic studies (Yang et al., 2011). Likewise, S100B is a useful progenitor marker, but it is also expressed in oligodendrocyte progenitors (Deloulme et al., 2004) and NG2 cells (Hachem et al., 2005). The pressing need to identify more markers of both rodent and human astrocytes to further investigate these cells under various conditions is evident. Ultimately, hPSC-derived astrocytes should be identified by astrocyte-specific functional outputs, but traditional readouts such as glutamate uptake and promotion of synaptogenesis are also functions of progenitor cells and other glial types to differing extents.

Unfortunately for researchers interested in generating purified clonal mature astrocytes as quickly as possible for functional studies, the prolonged developmental timeline of this heterogenous culture system mentioned above (approximately 4–6 months depending on the protocol used) is an inconvenience. The astrocyte differentiation process can be accelerated by prolonged treatment with gliogenic factors including CNTF, BMP, and LIF, or possibly by imposing epigenetic changes to occur (Gupta et al., 2012a). However, these treatments could have some effect upon disease phenotypes or even mask them. To date, no systematic comparison has been made of astroglial cells derived from the same starting material with differing methods. For a more temporally pure population it may be possible to clonally culture and expand a single cell, although proliferation of human astrocytes appears to be density dependent (Krencik, pers. observation). Other possible options are to cell-sort progenitors based on distinct cell type markers as has been done with CD44 (Yuan et al., 2011) or to transform fibroblasts directly into astrocytes using transcriptional codes similar to what has been done for the transdifferentiation of human fibroblasts to neurons (Pang et al., 2011). Regardless of the techniques used, methods should be considered that most accurately produce the cell type of interest for disease studies and detailed method descriptions should be provided for repeatability and data comparison between research groups.

It is understood in quantum mechanics that any observation of a system will have some change on the system itself, thus affecting the final measurement. In cellular neuroscience, we obviously disturb the cellular order by designing simplified models outside of the natural state. The major shortcoming of using hPSC-derived neural cells (and primary cultures from rodent or human origin) for disease modeling is the changes that occur in culture, which is usually a more stressful environment compared to the normal *in vivo* nervous system. Yet, the use of cultured cells confers many advantages including cell type purity, control of environmental factors, and easy access for experimentation. Astrocytes were first cultured from early postnatal rodent brain by taking advantage of their adhesiveness, survivability, and enhanced growth rates compared to other neural types while cultured in serum conditions (McCarthy and de Vellis, 1980) or after subsequently switching to serum-free defined conditions (Morrison and de Vellis, 1981). Serum varies between batches and leads to changes in morphological and proliferative properties, and therefore it is not suggested for use with primary astrocyte cultures; yet many studies are still conducted in serum containing conditions for its ease of use. More recently, an immunopanning technique has been designed for a rapid serum-free purification, revealing major gene expression changes in the presence of serum (Foo et al., 2011). High serum also affects hPSC-derived astrocytes in their morphology, adhesiveness, proliferation, and usually results in the expansion of non-neural contaminants at early differentiation stages (Krencik, pers. observation). Another stressor is that cells are typically cultured at higher oxygen levels than physiological conditions, which may impact progenitor proliferation and/or differentiation (Studer et al., 2000). The choice of culture media and additives will likely affect the differentiation process as well. For example, long term expansion of human NSCs requires the presence of growth factors such as EGF and FGF2 (Caldwell et al., 2001), but variability in the starting concentrations and metabolism of these factors, and the time between media replacement, will likely be inconsistent between different cultures and researchers. One advantage of using these growth factors is that for RASopathy studies the presence of these factors directly activates the Ras/MAPK pathway through receptor tyrosine kinase receptors, thus this pathway is chronically stimulated and may expose disease-related phenotypes. Taken together, although these culture factors may produce a system that is dissimilar to the natural environment, the stressful conditions may accelerate or expose disease phenotypes that normally do not appear until adulthood in humans and evolve over years such as neurodegenerative diseases. Even though all neurodegeneration-related phenotypes will be unlikely to be present in this relatively short-term culture system, cellular phenotypes have been observed with Parkinson's disease (Devine et al., 2011; Nguyen et al., 2011) and ALS cellular models (Bilican et al., 2012). It is important to note that even after astrocytes are generated, semi-purified, and prepared for experimentation using the method of choice, the issue still remains as to whether the final product is the best functional model for the disease of interest.

## How to traverse this diverse multiverse

For modeling region-specific diseases *in vitro*, the most relevant cell subtype to generate is one that displays similar dysfunctional properties as cells in the natural disease system. Precise directed differentiation of specific neuronal subtypes from hPSCs is increasingly attainable due to the ease of subtype identification using expression of neurotransmitter-related factors as markers; for example, observing the presence of choline acetyltransferase in motoneurons (Li et al., 2005), tyrosine hydroxylase in dopaminergic neurons (Zeng et al., 2004) or GABA in striatal interneurons (Aubry et al., 2008). Though astrocytes are known to secrete various gliotransmitters including ATP and D-serine, these are unlikely ideal markers because questions still remain about the physiological role of gliotransmission due to possible experimental artifacts including astrogliosis (Agulhon et al., 2012), and whether these gliotransmitters are variably expressed between subtypes. However, it is known that astrocytes display heterogenous enzymatic activities (Hansson, 1984) and responses to neurotransmitters in regionally distinct subtypes, correlating with their adjacent neuronal subtypes (Matyash and Kettenmann, 2010; Oberheim et al., 2012). Since hPSC-derived NSCs can be regionally specified at the neuroepithelia stage by the application of morphogens and then further matured into neurons or glia that maintain this identity (Liu and Zhang, 2011), regional distinctions that can be measured *in vivo* may also occur *in vitro* if these functions are endowed through intrinsic mechanisms. For example, the promoter activity for the astrocyte specific glutamate transporter Glt-1 is lower in spinal cord astrocytes compared to those in the brain (Regan et al., 2007) and the inward rectifying potassium channel Kir4.1 protein is more abundant in ventral spinal cord compared to dorsal regions (Olsen et al., 2007). Morphology and proliferation rates are also region-specific (Emsley and Macklis, 2006). *In vitro*, it has been shown that astrocytes prepared from different regions exhibit differential effects upon neurons including neuronal outgrowth (Qian et al., 1992), dendritic arborization (Le Roux and Reh, 1995), and differentiation (Castelo-Branco et al., 2006). Whether these functional distinctions can be recapitulated by hPSC-derived astrocytes specified to distinct subtypes is still unknown. Together, some of these specific markers and functions may be useful for identification after directed differentiation of hPSCs toward the distinct type of interest.

Does astrocyte regional heterogeneity have relevance for disease studies? *In vivo*, the degree of astrocytic responses in disease states may be due to both intrinsic diversity and responses to the local environment. For example, midbrain astrocytes may respond differentially to changes in dopamine levels in early stages of Parkinson's disease due to variable MAO-B levels (Mallajosyula et al., 2008; Vaarmann et al., 2010) and spinal cord astrocytes may not be able to properly reduce glutamate levels during ALS-induced excitotoxicity due to a low level of Glt-1 (Regan et al., 2007). This heterogeneity also has relevance for regenerative medicine. Midbrain astrocytes can secrete neurotrophic factors that protect dopaminergic neurons from degeneration including GDNF and CDNF (Lin et al., 1993; Lindholm et al., 2007), although these factors are also expressed in other regions. Developmental studies have revealed that astrocyte functional diversity may at least partially depend on their domain of origin. For example, dorsally and ventrally located astrocyte progenitors differentially express the guidance cues Slit1 and Reelin (Hochstim et al., 2008) and the extracellular matrix protein tenascin C (Karus et al., 2011). It is unknown whether these differences also depend on environmental cues, though it is interesting that astrocytes continue to occupy their distinct subregional domains determined in development and do not migrate to other domains after injury or depletion of adjacent cells (Tsai et al., 2012). With regards to RASopathy modeling, astrocytomas in NF1 predominantly occur around or near the optic nerve, thus, it may be most relevant for cancer studies to direct hPSCs to the optic stalk neuroepithelium, the probable source of optic nerve astrocytes (Horsburgh and Sefton, 1986). Though it is unknown why gliomas preferentially occur in the optic nerve, there is evidence of heterogenous astrocyte expression of NF-1 (Yeh et al., 2009), region specific effects of NF1 on astrocyte differentiation (Lee da et al., 2010), subtype astrogliosis (Rizvi et al., 1999) and differential responses from the local environment (Simmons et al., 2011) which may all play some role. As another example, abnormal vision is very common in Noonan syndrome including refractive errors (Sharland et al., 1992), therefore astrocytes differentiated from hPSC-derived retinal and optic nerve progenitor cells (Lamba et al., 2006; Meyer et al., 2009; Nakano et al., 2012) may be a promising candidate for investigation.

Another important variable to consider includes the maturation state of the astrocyte. hPSC-derived astrocytes are likely a mix of NSCs and astroglial progenitors during the differentiation process as described above (Figure 1), making measurements of mature functions variable in this mixed culture system. At the genomic level, astrocytes isolated at different stages of development differ in expression of numerous genes (Cahoy et al., 2008). Even after further maturation of hPSC-derived astrocytes in prolonged culture, these cells are likely immature compared to human adult astrocytes since they have been differentiating for only a few months and have not received signals from neurons that are known to affect astrocyte-specific proteins (Stipursky et al., 2012). For example, the Glt-1 protein remodels its localization near neighboring neuronal synapses during development (Benediktsson et al., 2012). Conversely, immature astrocytes may be better suited for studying synaptogenesis because immature astrocytes, but not mature, secrete the synaptogenic factor thrombospondin (Christopherson et al., 2005). It has also been revealed that developing and adult astrocytes functionally differ based on differential expression of glutamate receptors (Sun et al., 2013). Importantly, the key factor in modeling disease is that after generating the most relevant cell of interest, it would be critical to determine whether these cultured hPSC-derived astrocytes are exhibiting distinct responses to specific disease paradigms or whether they are only displaying generic responses independent of the stress paradigm (i.e., increases in oxidative stress, ER stress, or cell death), though there is now evidence of specific transcriptional changes that occur depending on the type of astrocyte stimulus (Lavisse et al., 2012; Zamanian et al., 2012).

## Extracellestial contact

Even though cultured astrocytes imperfectly recapitulate cells in the brain environment as discussed above, the use of cultured primary astrocytes has been instrumental in elucidating their influence on other cell types in both normal and diseased conditions, as thoroughly reviewed elsewhere (Lange et al., 2012). Importantly, the use of human-specific astrocytes from hPSCs may uncover unique phenotypes, including those that are known to exist *in vivo* (Oberheim et al., 2009), which can be masked in non-human backgrounds or otherwise difficult to measure due to limited resources of human tissue. In the case of RASopathies, immunochemical analysis with human NF1 brains has revealed an increase of astrogliosis, though how this contributes to neuronal abnormalities is unknown (Nordlund et al., 1995). Structural MRI studies of Costello syndrome brains usually uncover macrocephaly, ventriculomegaly, and Chiari 1 malformation which suggests an increase of astrocyte progenitor number (Gripp et al., 2010) similar to what has been observed in mouse models. Another experimental option for human cellular studies is use of human fetal NSCs for analysis. Recent use of this system has revealed that the Ras/MAPK pathway may be dysregulated in both Fragile X and Down syndrome genetic backgrounds (McMillan et al., 2012); suggesting this pathway plays a major disease role outside of RASopathies. In light of these issues, the use of hPSC-derived astrocytes would be a convenient human specific system to study intrinsic changes in RASopathy genetic backgrounds including astrogliosis and/or proliferation. Besides intrinsic cellular changes, disease modeling can shed light on what factors diseased astrocytes bestow upon other cells types. Because it is difficult to separate the effects of specific cell types *in vivo*, the system can be used for identifying these factors in either coculture, with use of astrocyte conditioned media (ACM), or transplantation studies (Figure 1).

Astrocytes receive and send signals to numerous cell types throughout the nervous system; thus simplified coculture systems may be a means to reveal important signaling components while optimizing additional extrinsic factors. One major route of communication apt for examination is between the brain vasculature system and astrocytic end feet (termed the gliovascular unit) which plays diverse roles in normal and diseased states. These functions include the coupling of neuronal activity to blood flow regulation and maintenance of the blood-brain-barrier (BBB) (Iadecola and Nedergaard, 2007; Kovacs et al., 2012). In order to study the effect of astrocytes on BBB formation and maintenance, numerous direct and indirect co-culture systems have been designed for studies with brain endothelial cells, as extensively reviewed elsewhere (Naik and Cucullo, 2012; Lippmann et al., 2013), although the specific molecules they provide is unclear. In the other direction, cultured endothelial cells induce astrocyte differentiation possibly through LIF secretion (Mi et al., 2001). Astrocytes also play major roles in synaptogenesis and synaptic plasticity (Allen and Barres, 2005; Eroglu, 2009; Barker and Ullian, 2010). Astrocyte-neuronal cocultures and/or ACM have been used to uncover numerous synaptogenic factors released by astrocytes which include cholesterol (Mauch et al., 2001), TNFα (Beattie et al., 2002), thrombospondins, (Christopherson et al., 2005), Hevin, Sparc (Jones et al., 2011; Kucukdereli et al., 2011), and glypicans (Allen et al., 2012). How these and other factors that alter synaptic plasticity [including extracellular matrix molecules and cytokines (Wiese et al., 2012)] change during development and disease in human cellular backgrounds are unclear. One method to investigate these changes may be quantitative secretomics via mass spectrometry as has been conducted with mouse astrocytes (Dowell et al., 2009; Jha et al., 2012). Simplified coculture studies between differing cell types may lead to discovery of drug targets to inhibit or activate these specific signaling pathways, as has been conducted in the case of a thrombospondin receptor (Eroglu et al., 2009).

Ultimately, cell–cell interactions should be studied in a more natural environment for proper cell–cell communication to limit astrogliosis and provide the human astrocytes with extracellular matrix factors (Figure 1). One potential experimental model system includes hippocampal slice cultures, which have been previously used from human epilepsy patient tissue to measure functional changes in astrocytes (Hinterkeuser et al., 2000). Since live human brain tissue is rarely available for other neurological diseases, it would be more feasible to inject or overlay hPSC-derived astrocytes into rodent slice cultures to allow functional integration followed by characterization with electrophysiological recordings, as has been conducted with mouse stem cell-derived glial progenitors (Scheffler et al., 2003; Husseini et al., 2008). For example, this coculture system has been utilized with hPSC-derived neurons in order to measure their resultant neuronal orientation and differentiation over time (Shi et al., 2012). Better yet, cells can be directly transplanted into a live animal for long term functional integration as has been previously conducted (Weick et al., 2011). What functional measurements would be informative after engraftment of human astrocytes into the rodent nervous system? Potential measurements could include the response of diseased astrocytes to synaptic activity via glutamate uptake measurements (Bergles and Jahr, 1998) or while calcium imaging during different experimental paradigms (Duffy and MacVicar, 1995; Torres et al., 2012), interactions with brain vasculature (Mulligan and MacVicar, 2004; Krencik et al., 2011), or the response of adjacent neurons after astrocyte stimulation with optogenetic tools (Figueiredo et al., 2011; Sasaki et al., 2012). Besides investigating questions about diseases, this system could also be used for regenerative medicine. For example, human astrocytes may be used as neuroprotective tools by transplanting them into a diseased system (Lepore et al., 2008) or as vehicles to deliver neurotrophic compounds (Drinkut et al., 2012).

## Conclusions

Astrocyte differentiation includes most of the same advantages and drawbacks that exist when generating heterogenous neural and non-neural cell types from hPSCs, although as described above they have additional technical challenges that include an extensive developmental timeline, limited cell specific tools, and sensitivity to stressful stimulations that leads to reactive astrogliosis. By summarizing these challenges and the best techniques with which to meet them (Table 1), this review can be used as an atlas to accordingly plan studies to produce the best model system possible, while keeping efforts of the researchers at a minimum. With careful preparation, functional analysis of astrocytes during disease studies can be conducted with high standards to account for cellular and temporal heterogeneity, although understandably most studies cannot address all the issues listed above. Typically, neurodevelopmental diseases such as RASopathies and neurodegenerative diseases have been modeled using neuronal cell types, but the roles of astrocytes in other less studied pathologies may also be targeted such as neuropathic pain (Watkins and Maier, 2003), sleep, and memory (Ben Achour and Pascual, 2012). Future technologies including high throughput screens, robotic apparatuses, computer models, and improved data analysis programs will undoubtedly increase the scale and accuracy of data collection/interpretation. Together with improved techniques to image and monitor healthy and diseased human astrocytes in culture or engrafted into a donor nervous system, the potential for glial studies in regenerative medicine and future disease discoveries can reach to the stars.

**Table 1:** **Atlas for using hPSC-astrocytes in normal and disease states**.

**Stages**	**Challenges**	**Recommendations**
hPSCs	Heterogeneity between lines	Use similar source material (cell type, age of donor, passage number, etc.)
		Use non-integrative reprogramming
		Generate control lines with pharmacological or genetic correction technology
Neuroepithelia/NSCs	Regional heterogeneity	Generate/select for CNS-specific cells instead of PNS neural crest
		Specify to distinct dorsal-ventral and anterior-posterior axis if needed
Astrocyte progenitors	Stressful culture environment	Use serum free conditions plus additives such as antioxidants
		Lower oxygen conditions, avoid acidity
		Limit stress during passaging
	Non-synchronous mixed culture	Cell sort if purification is desired
		Mature long term with factors such as CNTF to induce GFAP
		Remove adherent non-neural cells via astrosphere culture method (Krencik and Zhang, 2011)
Mature astrocytes	Identification of mature vs. immature	
	Identification of mature vs. reactive	Not yet well-defined. Conduct quantitative measurement of GFAP and other markers over time
	Functional characterization	Not yet well-defined
		Recommended assays include proliferation, synaptogenic studies with neuron cocultures, receptor/transporter stimulation followed by electrophysiological measurements or calcium imaging (Krencik et al., 2011), BBB formation/maintenance of endothelia
Engrafted astrocytes	Test for functional integration	Measure: Glutamate uptake after synaptic stimulation
		Calcium wave propagation between endogenous and engrafted human astrocytes
		Endfeet formation on blood vessels, constriction assay after stimulation
		Rescue a mouse disease model
Diseased states	Determine disease-specific phenotype	Assay for known disease-related phenotype in monoculture, coculture, or post-engraftment
		Profile at various levels for reactive signature
	Rescue the phenotype	Screen pharmacological or genetic (siRNA, etc.) methods

### Conflict of interest statement

The authors declare that the research was conducted in the absence of any commercial or financial relationships that could be construed as a potential conflict of interest.
